# Mitochondria Know No Boundaries: Mechanisms and Functions of Intercellular Mitochondrial Transfer

**DOI:** 10.3389/fcell.2016.00107

**Published:** 2016-09-28

**Authors:** Daniel Torralba, Francesc Baixauli, Francisco Sánchez-Madrid

**Affiliations:** ^1^Signaling and Inflammation Program, Centro Nacional Investigaciones CardiovascularesMadrid, Spain; ^2^Servicio de Inmunología, Instituto Investigación Sanitaria Princesa, Universidad Autonoma de MadridMadrid, Spain

**Keywords:** horizontal genetic transfer, tunneling nanotubes, mitochondrial diseases, extracellular vesicles, inflammation, DAMPs, exosomes, communication

## Abstract

Mitochondria regulate multiple cell processes, including calcium signaling, apoptosis and cell metabolism. Mitochondria contain their own circular genome encoding selected subunits of the oxidative phosphorylation complexes. Recent findings reveal that, in addition to being maternally inherited, mitochondria can traverse cell boundaries and thus be horizontally transferred between cells. Although, the physiological relevance of this phenomenon is still under debate, mitochondria uptake rescues mitochondrial respiration defects in recipient cells and regulates signaling, proliferation or chemotherapy resistance *in vitro* and *in vivo*. In this review, we outline the pathophysiological consequences of horizontal mitochondrial transfer and offer a perspective on the cellular and molecular mechanisms mediating their intercellular transmission, including tunneling nanotubes, extracellular vesicles, cellular fusion, and GAP junctions. The physiological relevance of mitochondrial transfer and the potential therapeutic application of this exchange for treating mitochondrial-related diseases are discussed.

## Introduction

Mitochondria regulate cellular homeostasis by controlling energy production, calcium signaling, cell metabolism and apoptosis. According to the endosymbiotic theory, mitochondria are derived from eubacteria that engaged in a symbiotic relationship with primitive host cells. They have maintained some of their ancestral bacterial characteristics, such as a double membrane, a proteome similar to the α-proteobacteria, and the ability to generate the majority of ATP from cells in the process of aerobic respiration or oxidative phosphorylation (OXPHOS) (Friedman and Nunnari, 2014).

Mitochondria are endowed with their own circular genome, which is 16.6 kb in length in mammalian cells. This mitochondrial DNA (mtDNA) encodes 13 subunits that are essential components of the electron transport chain, and a translational system made of two ribosomal RNAs and 22 tRNAs, required for the translation of these proteins (Gustafsson et al., 2016). During evolution, most of the mitochondrial ancestor genes have been progressively transferred to the nuclear genome, or lost; while mitochondria have acquired new components and functions from the host cell (Keeling and Palmer, 2008). A typical mammalian cell contains thousands of copies of the mitochondrial genome, which are organized in mtDNA-protein complexes known as nucleoids (Kukat et al., 2015). The mitochondrial genome copies of a given cell can be identical (homoplasmy), or contain more variants (heteroplasmy). Although it was generally assumed that healthy individuals exhibit homoplasmy for a single mtDNA genotype, next-generation sequencing has revealed the presence of low-level heteroplasmy in most tissues from healthy individuals (He et al., 2010; Payne et al., 2013). The source of this heteroplasmy is likely to be maternally inherited, as some of the low-frequency alleles found in an individual can also be found in the mother. They may also stem from somatic mutations that occur during tissue development and aging. In inherited mitochondrial DNA diseases, individuals are typically heteroplasmic and harbor both wild-type and pathogenic mtDNA molecules. Although, cells can tolerate a heavy load of pathogenic mutations, these will lead to disease when the mutation load surpasses a tolerable threshold, causing multisystemic diseases that specially affect organs with high energy requirements such as the brain, muscle, and heart. Additionally, many somatic mutations in mtDNA are related to a number of other pathologies such as cancer, cardiovascular diseases and neurodegenerative disorders, as well as the normal aging process (Wallace, 2013).

In mammals, mitochondria are transmitted to subsequent generations exclusively through the maternal lineage. Uniparental germline inheritance reduces the benefits of recombination arising from sexual reproduction, also facilitating the appearance of maternally inherited disorders when a significant amount of mtDNA mutations are transmitted to the offspring. To counteract this, the mtDNA population undergoes a restricted process of purifying selection during oogenesis, allowing only a small population of mtDNA molecules to be amplified and transmitted. Moreover, selective replication and degradation can expand a subpopulation of mtDNA molecules at the expense of others, leading to the rapid segregation of mtDNA variants within a few generations (Stewart and Larsson, 2014). During somatic cell proliferation, symmetric cell division implies that mitochondria and mtDNA are distributed between both daughter cells. Segregation of mitochondria and mtDNA involves mitochondrial fission, facilitated by the interaction of mitochondria with cytoskeletal and endoplasmic reticulum elements (Friedman et al., 2011; Lewis et al., 2016). Mitochondrial partitioning during symmetric division can be envisioned as a stochastic process in which mitochondrial subunits are distributed randomly between daughter cells. Conversely, recent findings suggest that asymmetric cell division differentially segregates young and old mitochondria, revealing the existence of active mechanisms that guide mitochondria partitioning between daughter cells (Katajisto et al., 2015).

Several mechanisms maintain the integrity of mitochondria and mitochondrial genome in post-mitotic cells. Constant cycles of fusion and fission promote the intermixing and the homogenization of mitochondrial proteins, lipids and DNA between discrete organelles within the same cell (Mishra and Chan, 2014). Consistent with this, cells lacking proteins involved in mitochondrial dynamics, e.g., mitofusins, show a striking heterogeneity among mitochondria in terms of protein and mtDNA nucleoid content, and membrane potential (Chen et al., 2010). This causes defects in the respiratory chain function and promotes the accumulation of mtDNA mutations. The selective elimination of mitochondria through mitophagy eliminates deleterious mitochondrial mutations in heteroplasmic cells, restoring ATP levels and mitochondrial functions (Suen et al., 2010; Dai et al., 2014; Valenci et al., 2015). However, increased mitochondrial biogenesis in heteroplasmic cells replenishes the amount of deleterious mtDNA (Gitschlag et al., 2016; Lin et al., 2016). Therefore, specific mechanisms regulate the stability and transmission of mitochondria and their genome during germline transmission and somatic cell proliferation. This is enabled by purifying selection and selective replication or degradation of mtDNA. In non-dividing cells, the dynamics of mitochondrial networks and their selective degradation or biogenesis maintains a healthy mitochondria repertoire.

Mitochondria have been recently shown to be horizontally transferred between mammalian cells, challenging current concepts of mitochondria and mtDNA segregation and inheritance. This transfer promotes the incorporation of mitochondria into the endogenous mitochondrial network of recipient cells, contributing to changes in the bioenergetic profile and in other functional properties of recipient cells, not only *in vitro* but also *in vivo*. Moreover, intercellular transfer of mitochondria involves the horizontal transfer of mitochondrial genes, which has important implications in the physiopathology of mitochondrial dysfunction. The intercellular transfer of mitochondria or their components, may also result in the initiation of stem cell differentiation, reprogramming of differentiated cells, or activation of inflammatory signaling pathways. Diverse structures mediate intercellular mitochondrial transfer. These include tunneling nanotubes (TNTs), microvesicles, mitochondrial ejection or cytoplasmic fusion, among others. Future research in this field is likely to open new avenues for the treatment of mitochondrial related disorders, e.g., transfer of healthy, or genetically corrected, mitochondria.

In this review, we discuss the relevance of mitochondrial transfer in physiological processes. Starting from an integrated view of the molecular mechanisms mediating intercellular mitochondrial transmission, we delve into the pathophysiological consequences of horizontal mitochondrial transfer. Finally, we discuss how intercellular mitochondrial transfer can be exploited as a new avenue for the treatment of mitochondrial-related diseases.

## Intercellular mitochondrial transfer

The existence of nanotubular structures mediating the transfer of many cellular constituents (Rustom et al., 2004) has demonstrated the widespread ability of mammalian cells to donate or receive organelles from other cells (Gerdes et al., 2007; Davis and Sowinski, 2008). Such transfer involves many different cell types *in vitro* and *in vivo*, including lymphocytes, neurons or cardiomyocytes. Organelle exchange represents a special form of intercellular communication that allows unidirectional or bidirectional transfer, not only of signals, small molecules or ions, but also of defined intracellular structures such as mitochondria, lysosomes, endosomal vesicles and plasma membrane components (Rogers and Bhattacharya, 2013).

The first evidence of functional mitochondrial transfer involved healthy mitochondria from human stem cells rescuing mitochondrial respiration in mitochondria-depleted recipient cells (Spees et al., 2006). Cells devoid of intrinsic mitochondrial function by mtDNA depletion through long-term ethidium bromide treatment, do not survive in standard media. However, when these cells were co-cultured with mesenchymal stem cells (MSCs) or fibroblasts that donated functional mitochondria, they acquired mitochondria and re-established aerobic respiration. The careful examination of mitochondrial and nuclear DNA polymorphisms in the rescued clones excluded cell fusion as the main mechanism of mitochondrial transfer, also confirming the stem cell origin of the transferred mtDNA (Spees et al., 2006). Transfer did not occur through the passive uptake of mitochondrial fragments or isolated organelles, but rather involved active processes such as the formation of TNTs and/or the vesicular transfer of mtDNA or mitochondrial fragments.

Other groups have demonstrated that the horizontal cell-to-cell transfer of mitochondria and mitochondrial genome can occur among many other mammalian cells *in vitro* and *in vivo* (Berridge et al., 2016). Mitochondrial transfer supports the exogenous replacement of damaged mitochondria, thereby rescuing mitochondrial defects (Hayakawa et al., 2016; Patananan et al., 2016). Most studies used stem cells as mitochondrial donors, but some others also used immortalized cells or primary cells from the same or different species (Berridge et al., 2016). Although, there are some concerns regarding whether intercellular mitochondrial transfer is a physiologically relevant process or it is only observed in cell culture conditions, recent evidence strongly supports that mitochondrial transfer does occur *in vivo* and may be involved in diverse pathophysiological situations, such as tissue injury and cancer progression (Islam et al., 2012; Ahmad et al., 2014; Tan et al., 2015; Hayakawa et al., 2016; Moschoi et al., 2016).

Intercellular mitochondrial transfer rescues injured cells from mitochondrial dysfunctions related with several models of cell survival associated to stress responses. MSC improve survival and restore cell damage by transferring their mitochondria through TNTs to EC or cardiomyocytes previously subjected to an *in vitro* model of ischemia-reperfusion, in which cells are cultured under glucose-oxygen deprivation and then reoxygenated (Liu et al., 2014; Han et al., 2016). Similarly, the transfer of mitochondria from MSC to human umbilical vein endothelial cells (HUVEC) previously subjected to *in vitro* ischemia-reperfusion, restores aerobic respiration in EC independently of cellular trans-differentiation or the release of paracrine factors. Notably, aerobic restoration is not reestablished in HUVEC when these are cultured alone or with MSCs containing dysfunctional mitochondria promoted by mtDNA depletion (Liu et al., 2014). Interestingly, phosphatidylserine exposure on injured endothelial cells acts as a “find-me” signal that triggers the emergence of tunneling nanotubes and guides them from MSC to injured HUVECs (Liu et al., 2014). Mitochondrial transfer from MSCs to lung epithelium also attenuates cigarette smoke-induced lung damage (Li et al., 2014), while transfer between MSC and innate immune cells via TNTs and microvesicle secretion enhances the capacity of alveolar macrophages to engulf invading bacteria in an *E.coli* pneumonia model (Jackson et al., 2016). Based on these results, it has been proposed that cell stress is required for organelle transfer. Mitochondrial transfer is triggered by an almost complete absence of mitochondrial function, such as mtDNA depletion or treatment with mitochondrial inhibitors, since transfer is not detected to cells harboring pathogenic mutations that partially affect mitochondrial function (Cho et al., 2012; Wang and Gerdes, 2015). An intriguing question pertains to the degree of cellular damage required to initiate intercellular transfer of functional mitochondria. We also ponder about the mechanisms by which the healthy cell detects the degree of metabolic shutdown of the stressed cell and makes a “judgment call” to restore the functionality of the stressed cell, instead of permitting apoptosis to remove the damaged cell. This would be of particular interest in non-replaceable cell lineages such as adult neurons. Accordingly, the transfer of mitochondria from astrocytes to neurons has a neuroprotective effect after stroke (Hayakawa et al., 2016).

**Figure 1:**
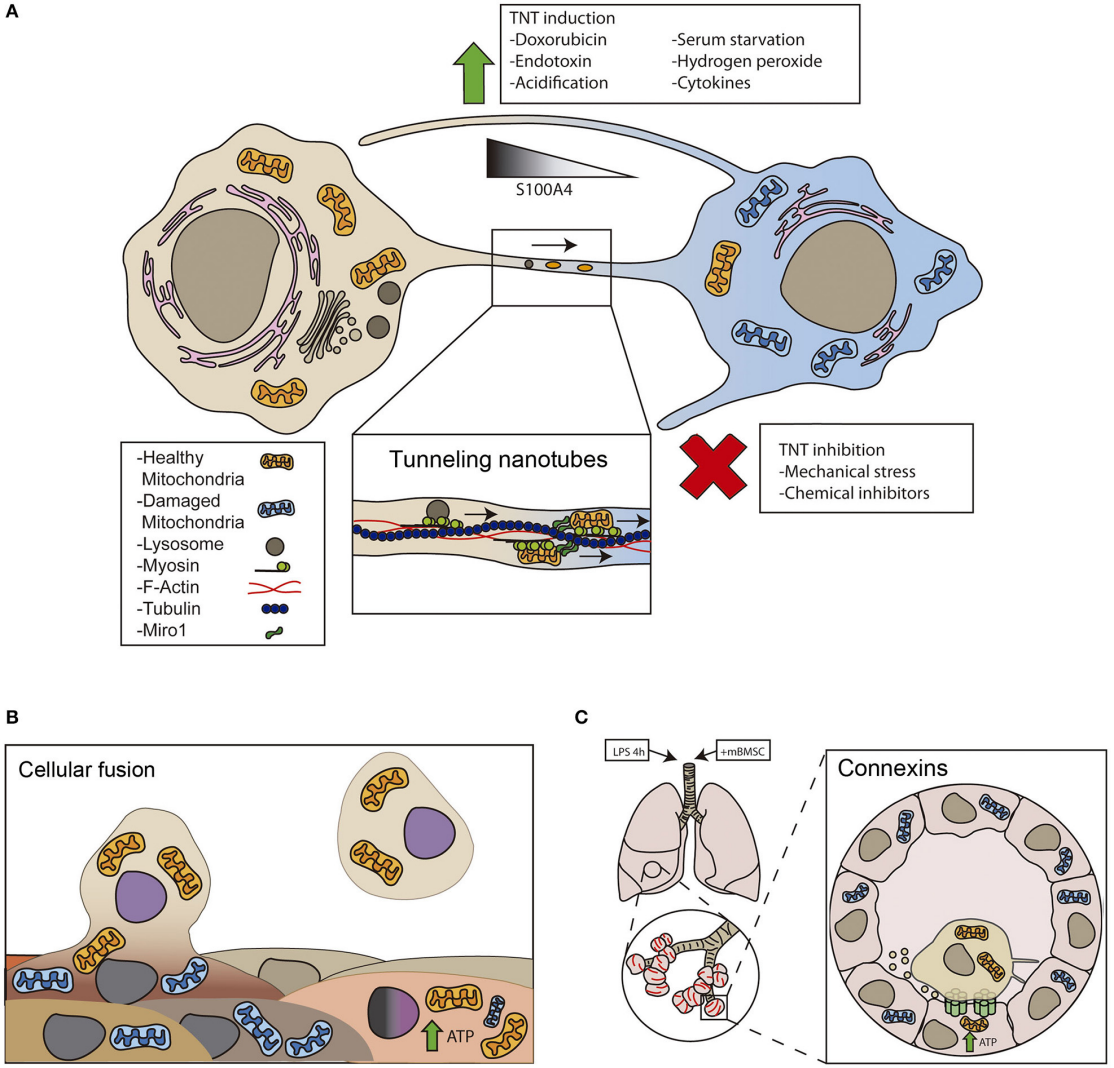
**Cellular mechanisms of intercellular mitochondrial transfer. (A)** *Tunneling nanotubes* (TNT) are long ultrathin structures with diameters ranging from 50 to 200 nm and a length that allow organelle transfer between two spatially separated cells. TNTs contain cytoskeletal elements such as actin and microtubules depending on the cell type. Myosin is a fundamental protein for the organelle transfer, a process where high rates of ATP consumption are needed. Rho GTPases play an important role in organelle transfer through TNT. Miro1 and microtubules have been involved in the transfer of mitochondria upon injury (Ahmad et al., 2014). **(B)** *Cell fusion* is a process, in which two independent cells fuse their membranes and share organelles and cytosolic compounds, however nuclei remain intact. Cell fusion can be transitory and therefore lose their integrity transiently or complete, where cells will share cytoplasm continuously, for example in muscle cells. It has been shown that after myocardial infarct stem cell therapy can lead to a better recovery and it is proved that partial or total fusion events can happen between cardiomyocytes and stem cells (Oh et al., 2003). By this mechanism mitochondrial respiration can be restored and this could be an indicative marker for cardiac regeneration after infarct. **(C)** *GAP junctions*. LPS treatment increases connexin 43 expression in alveolar cells. Connexins facilitate the attachment of BMSC to the alveolar epithelial cells, and produce vesicles and nanotubes to share mitochondria with the damaged cells protecting against acute lung injury (Islam et al., 2012).

Mitochondrial transfer seems to be involved in stem cell-triggered repair of damaged cells (Plotnikov et al., 2008). Mitochondrial transfer from stem cells to lung epithelial cells and endothelial cells is an important mechanism by which MSC exert their protective effects in several animal models of lung diseases. A seminal study demonstrated that transfer of intact mitochondria can contribute to tissue repair *in vivo* (Islam et al., 2012). In that study, bone marrow-derived stem cells (BMSCs) infused to the trachea of lipopolysaccharides (LPS)-treated mice attached to injured alveolar epithelial cells by a mechanism involving connexins (Figure 1). Connexin oligomerization forms channels or GAP junctions that connect two cells, allowing the transfer of only small metabolites, ions or small RNAs. Connexins, and specifically connexin-43, regulate intercellular mitochondrial transfer by promoting the attachment of BMSCs to LPS-injured alveolar epithelial cells, leading to the formation of nanotubes and microvesicles that mediate mitochondrial transfer. Mitochondria acquisition by the alveolar cells triggered the restoration of ATP levels and increased the secretion of pulmonary surfactant. Silencing of RISP, a subunit of the mitochondrial complex III, in BMSCs reduced their capacity to restore ATP levels in recipient alveolar epithelial cells, avoiding protection from LPS-injury (Islam et al., 2012). These results implicated mitochondrial transfer as a driver of the *in vivo* benefits of mesenchymal stem cell therapy in models of acute lung injury and other inflammatory diseases, enhancing cellular bioenergetics and improving organ function. A follow-up study in mice provided a molecular mechanism to potentially increase the therapeutic efficiency of MSC by modulating the extent of mitochondrial transfer (Ahmad et al., 2014). This study described that the levels of Miro1, a protein connecting mitochondria to cytoskeletal motor proteins, regulate the efficiency of intercellular movement of mitochondria. Hence, overexpression of Miro1 in MSC increased mitochondrial transfer from stem cells into stressed epithelial cells via intercellular TNTs, attenuating epithelial cell apoptosis and reducing inflammatory cell infiltration, collagen deposition and mucus hypersecretion in the lungs. Overall, Miro1 overexpression improves the therapeutic efficiency of mitochondrial transfer across multiple models of asthma *in vivo* and *in vitro* (Ahmad et al., 2014).

Cancer cells opportunistically acquire mitochondria to optimize or repair their metabolic machinery. In this regard, horizontal transfer of mtDNA from host cells to tumor cells with compromised respiratory function has been described (Tan et al., 2015). Tumor cells devoid of mtDNA, a model of extreme mtDNA damage, acquire mtDNA from surrounding donor healthy cells, repairing the transcriptional and translational activity of mtDNA-encoded genes and triggering a substantial recovery of mitochondrial respiration, as well as tumor initiation efficacy and metastasis capacity. A major source of healthy mitochondria in these contexts are stromal cells, which can modulate the phenotype of the tumor by direct mitochondria transfer. Cancer cells and stromal cells communicate through TNTs, and mitochondrial transfer via TNTs from endothelial to cancer cells enhances their chemoresistance to doxorubicin *in vitro* (Pasquier et al., 2013). In addition, mitochondrial transfer from bone marrow mesenchymal cells to acute myelogenous leukemia cells *in vivo* confers chemoresistance and survival advantage (Moschoi et al., 2016). These findings highlight the requirement of mitochondrial respiration for tumorigenesis and metastasis, and support mitochondrial transfer as a mechanism for the efficient metabolic adaptation of tumor cells to hostile microenvironments (Tan et al., 2015).

### Mitochondrial transfer beyond cell rescue

Intercellular organelle transfer participates in cell differentiation and/or de-differentiation (Plotnikov et al., 2008, 2010). Human adipose-derived or bone marrow-derived stem cells can reprogram differentiated mouse cardiomyocytes into a progenitor-like state. This process involves partial cell fusion and mitochondrial transfer. Deprivation of mtDNA in stem cells dramatically decreased the efficiency of the reprogramming process. Although, mtDNA depletion may affect cell stemness and the ability to donate mitochondria, these results suggest that mitochondrial transfer from stem cells to adult cardiomyocytes helps their conversion to the progenitor state through metabolic reprogramming (Acquistapace et al., 2011). Stem cell interaction with other cell types does not necessarily result in actual differentiation, but it may rather initiate a proliferative response, as observed during mitochondrial transfer from vascular smooth muscle cells to MSCs (Vallabhaneni et al., 2012).

The release of mitochondria or mitochondrial components into the extracellular space has important immune consequences. After an episode of acute injury, release of mitochondrial components into the extracellular medium and bloodstream may trigger potent pro-inflammatory responses. In fact, mitochondrial components can be recognized by cells of the immune system as damage-associated molecular patterns (DAMPs) (Zhang et al., 2010; Galluzzi et al., 2012; West et al., 2015). Different mitochondrial proteins such as TFAM, together with other mitochondrial DAMPs like N-formyl peptides, mtDNA, cardiolipin or extracellular ATP, robustly activate the inflammatory response (Weinberg et al., 2015).

Mitochondrial DNA induces inflammation when found beyond mitochondrial membrane boundaries (Zhang et al., 2010). Mitochondrial DNA injected directly into mice joints causes inflammation and arthritis (Collins et al., 2004). Moreover, mitochondrial DNA that escapes autophagy leads to Toll-like receptor (TLR) 9-mediated inflammatory responses in a cell-autonomous manner (Oka et al., 2012). When abnormally found in the cytosol, mtDNA triggers NRLP3-inflammasome activation and STING-dependent antiviral signaling (Shimada et al., 2012; West et al., 2015), which ultimately leads to cell death. Neutrophils release to the extracellular environment oxidized mitochondrial nucleoids that activate the interferon response in pathological situations such as human systemic lupus erythematosus (Caielli et al., 2016), whereas activated platelets release into the bloodstream entire mitochondria or mitochondria encapsulated in microparticles to boost the inflammatory response (Boudreau et al., 2014). Interestingly, mtDNA released by eosinophils or basophils contribute to inflammatory responses creating DNA net traps to fight against invading pathogens (Yousefi et al., 2008; Morshed et al., 2014).

Mitochondria-dependent activation of the inflammatory response has important consequences to understand intercellular mitochondrial transfer. In this regard, innate immune cells target cells containing allogenic mtDNA (Ishikawa et al., 2010). Such recognition mechanism, displayed by natural killer cells and dendritic cells, is not yet understood, but it is likely to control the extent of intercellular mitochondrial transfer in pathophysiological conditions. As a consequence, careful modulation of immune surveillance mechanisms in recipient cells could be a potential strategy to boost exogenous mitochondrial donation for therapeutic purposes. Consistent with this view, MSC secrete microvesicles containing depolarized mitochondria that are engulfed by macrophages in the stem cell niche (Phinney et al., 2015). Together with microvesicles, MSC secrete exosomes that inhibit TLR signaling and macrophage activation, thereby promoting specific tolerance to the transferred mitochondria. Also, mitochondrial transfer by MSCs may enhance their own survival by unloading partially depolarized mitochondria into more professional phagocytic cells such as macrophages (Phinney et al., 2015).

A similar process of transcellular degradation of transferred mitochondria (“transmitophagy”) has been observed in neurons at the optic nerve head (ONH). Axonal protrusions and evulsions within the ONH contain mitochondria that are shed from neurons and degraded by the lysosomes of neighboring astrocytes (glial cells) (Davis et al., 2014). This process accounts for the majority of mitochondrial turnover, rather than mitophagy in the ganglion cell soma. The most likely reason for this mechanism is that it is energetically taxing to transport axonal mitochondria back to the soma for degradation. The system thus takes advantage of the presence of a subset of astrocytes with extreme phagocytic activity on the ONH (Davis and Marsh-Armstrong, 2014). In agreement, astrocytes can phagocytose whole synapses (Chung et al., 2013). A similar autophagy-assisted phagocytosis is also involved in the turnover of photoreceptor outer segments by retinal pigment epithelial cells (Kim et al., 2013). Although, transmitophagy is a relatively novel process in the nervous system, similar transcellular degradation of mitochondria occurs in gametes upon fertilization, in which sperm mitochondria are actively degraded by oocytes by the same molecular machinery used in autophagy (Al Rawi et al., 2011; Sato and Sato, 2011).

### Horizontal gene transfer

Although widely observed in prokaryotes, horizontal gene transfer (HGT) is in general a rare phenomenon in metazoans. However, HGT in eukaryotes might be less rare than previously anticipated (Crisp et al., 2015), and it has likely contributed to biochemical diversification during animal evolution. The horizontal transfer of genetic material between species of *Saccharomyces* has been postulated during the evolution of modern yeast species (Marinoni et al., 1999). Likewise, asexual bdelloid rotifers have experienced extensive HGT from non-metazoan genes (Gladyshev et al., 2008), which might have compensated the lack of genetic heterogeneity due to the absence of sexual recombination (Eyres et al., 2015). One interesting example is the horizontal transfer of entire genomes via mitochondrial fusion in the angiosperm *Amborella*. Specifically, *Amborella* mtDNA includes a diverse collection of foreign sequences corresponding to about six genome equivalents of mtDNA acquired from mosses, angiosperms and green algae (Rice et al., 2013).

One of the first evidences of *in vivo* horizontal mitochondrial gene transfer was found in a transmissible canine venereal tumor (CTVT), which is a highly adapted form of cancer that is transmitted as an allograft during mating of feral dogs. Phylogenetic evidence supports that CTVT cells periodically acquire mitochondria from their hosts to support long-term survival. Gene transfer events might rescue CTVT mitochondrial function, allowing the tumor to overcome the high mutation rate that would promote the accumulation of deleterious mutations in their own mitochondria (Rebbeck et al., 2011).

The intercellular exchange of mtDNA for the maintenance of heteroplasmy in cells has been recently proposed (Jayaprakash et al., 2015). Heteroplasmy is maintained in individual daughter cells over multiple cell divisions, which suggests the existence of active mechanisms that counteract mtDNA drift toward homoplasmy. Using a novel method to enzymatically purify mtDNA, the authors demonstrate the intercellular transfer of mtDNA between cell lines with distinct mtDNA haplotypes in co-culture experiments (Jayaprakash et al., 2015), raising the intriguing possibility that horizontal mtDNA exchange may regulate heteroplasmy *in vivo*.

## Cellular structures mediating intercellular mitochondrial transfer

The molecular and signaling mechanisms by which cells containing dysfunctional mitochondria acquire mitochondria from other cells, and how this process is regulated remain unclear. Cells likely possess mechanisms to trigger organelle exchange in response to injury signals emanating from the recipient cell. However, the molecular cues that initiate such crosstalk are still unidentified. Tunneling nanotubes are seen so far as the main cellular structure that mediates intercellular mitochondrial transfer. Other mechanisms have been proposed, including membrane microvesicles, cell fusion or mitochondrial ejection. Transfer through these diverse structures may result in different functional outcomes for the recipient cells, i.e., functional mitochondrial acquisition, immune activation or transmitophagy. Thus, a clear understanding on the mechanisms mediating mitochondrial transmission will shed light on how mitochondrial transfer is regulated and can be exploited for therapeutic purposes.

### Tunneling nanotubes

Tunneling nanotubes (TNTs) form between different cell types *in vitro* and *in vivo* (Figure 1). Tunneling nanotubes facilitate the selective exchange of organelle or membrane vesicles and small soluble cytoplasmic and membrane molecules. The establishment of a nanotube begins with the formation of a filopodium-like membrane protrusion that retracts after reaching the receptor cell, leaving an ultrafine structure that is separated from the substrate. TNTs appear to be essential for effective mitochondrial transfer, since impairing TNT formation with chemical inhibitors or mechanical stress reduces mitochondrial exchange, whereas inhibition of phagocytosis, endocytosis or exocytosis have little or no effect (Bukoreshtliev et al., 2009). Transfer of mitochondria through TNTs is usually unidirectional, from the cell that initiated the formation of TNT to the receptor cell (Rustom et al., 2004; Bukoreshtliev et al., 2009). However, bidirectional transfer has also been reported (He et al., 2011).

Certain types of stress agents increase TNT formation and mitochondrial transfer. Doxorubicin treatment increases mitochondrial transfer from endothelial progenitors to mature endothelial cells challenged with doxorubicin (Yasuda et al., 2010), while ethidium bromide treatment enhances mitochondrial transfer from MSC to osteosarcoma-treated cells (Cho et al., 2012). Similarly, multipotent mesenchymal stem cells transfer mitochondria to lung epithelium cells treated with cigarette smoke (Li et al., 2014). *In vivo*, acute lung injury promoted either by rotenone or TNF-treatment is required for the transfer of mitochondria from stem cells to lung epithelial cells (Ahmad et al., 2014). In general, mitochondrial damage is the main trigger of TNT-based mitochondrial transfer (Figure 1).

Other conditions that favor TNT formation are serum starvation and hydrogen peroxide. These conditions activate p53 and nanotube formation in hippocampal astrocytes and neurons (Wang et al., 2011). Similarly, hyperglycemic or acidified medium, as well as cytokines that stimulate epithelial-to-mesenchymal transition, increase mitochondrial transfer through tunneling nanotube formation (Lou et al., 2012). Therefore, stress can induce TNT formation, but further research is required to understand how TNT-mediated mitochondrial transfer is regulated. Notably, the protein S100A4 and its receptor guide the direction of nanotube growth (Sun et al., 2012). In stressed cells, p53 activates caspase-3 to cleave S100A4, creating a gradient in which the TNT initiating cell has the lower levels of the protein (Sun et al., 2012).

### Extracellular vesicles

**Figure 2:**
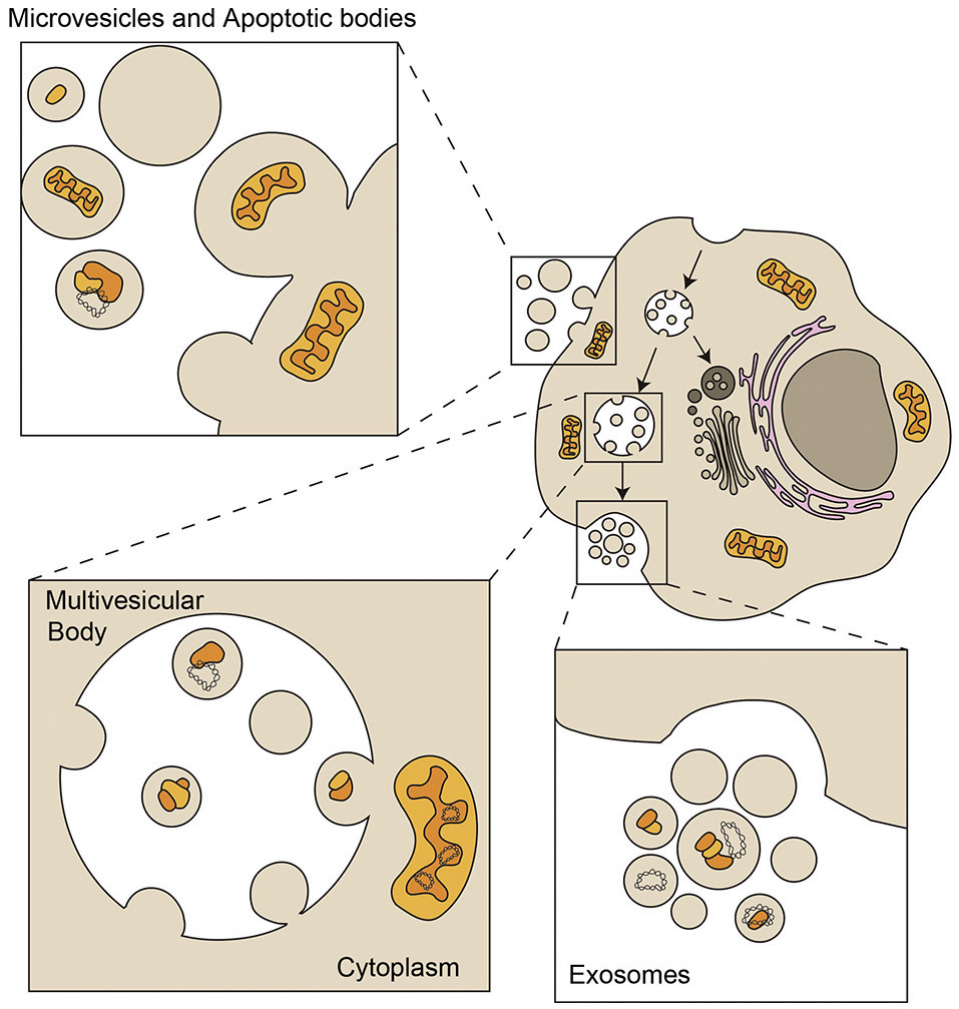
**Loading of mitochondrial components in Extracellular Vesicles**. Exosomes are small vesicles from 50 to 150 nm with an endocytic origin. The inward budding of late endosomes or multivesicular bodies (MVB) forms intraluminal vesicles that are released into the extracellular environment as exosomes through fusion of the MVB with the plasma membrane. Microvesicles, also called ectosomes, shed directly from plasma membrane and are very heterogeneous in size and composition. Apoptotic bodies are larger than 1 μm and are released by apoptotic cells that shed parts of their cytoplasm and organelles surrounded by plasma membrane (Mittelbrunn and Sánchez-Madrid, 2012). Extracellular vesicles can contain mitochondrial fragments which include mitochondrial proteins and mtDNA (depicted in yellow and orange). Bigger particles can be loaded with full functional mitochondria (Phinney et al., 2015).

Almost every cell type secretes a heterogeneous population of vesicles to the extracellular medium. This group of vesicles, ranging from 40 to 1000 nm, is referred to as extracellular vesicles (EVs). EVs can be divided into microvesicles, exosomes and apoptotic bodies, depending on their origin, size and molecular composition (Figure 2). EVs are vehicles of intercellular communication in a number physiological and pathological processes (Mittelbrunn and Sánchez-Madrid, 2012), and can be used as biomarkers of health and disease (Pitt et al., 2016).

Mitochondrial components have been detected in EVs, although the mechanisms by which mitochondrial proteins and mtDNA are loaded in the diverse EVs are still unknown. Exosomes, with a size ranging 40–150 nm, contain genetic material, mostly small RNAs (Villarroya-Beltri et al., 2014), but genomic and mitochondrial DNA have also been detected (Guescini et al., 2010a,b; Kahlert et al., 2014; Thakur et al., 2014). Larger EVs can contain entire mitochondrial particles and mtDNA, as observed in mesenchymal stem cells and astrocytes (Phinney et al., 2015; Hayakawa et al., 2016). These cells produce EVs with regenerative potential in several tissues under damaging conditions (Khan et al., 2015; Nakamura et al., 2015; Ong and Wu, 2015). Whether mitochondrial material contained in these vesicles participates in the regenerative potential of stem cells is yet to be shown.

EVs participate in intercellular mitochondrial transfer (Spees et al., 2006; Islam et al., 2012; Jayaprakash et al., 2015). Horizontal transfer of mitochondria occurs through the transfer of either mitochondria-derived vesicles or intact mitochondria. To date, the mechanisms of intercellular transfer of free mtDNA across both mitochondrial inner and outer membranes and the plasma membrane remain elusive. Also, the transfer of whole mitochondrial particles is the most likely event mediating rescue of mitochondrial function through EVs during intercellular mitochondrial transfer, in which some cells would acquire a few mitochondria and replicate their mtDNA.

### Cell fusion

Cell fusion is a process in which two independent cells fuse their membranes and share organelles and cytosolic compounds, while their nuclei remain intact. Permanent cell fusion result in cells that share the cytoplasm and possess unique karyotypes, while partial cell fusion allows transient, but direct, intercellular communication and exchange of multiple protein complexes and even subcellular organelles, including mitochondria. Fusion is a rare event in developed eukaryotic organisms, and a strict regulation of fusogen proteins and their receptors expression ensures that it only occurs under specific conditions (Aguilar et al., 2013).

Adult and embryonic stem cells have been reported to fuse with cardiomyocytes, hepatocytes and neurons, which might contribute to the differentiation, plasticity, or maintenance of these cell types (Alvarez-Dolado et al., 2003). Injury and inflammation may drive cell fusion in target organs. Cells from the myeloid and lymphoid lineage can fuse in low rates with different tissues in response to injury or inflammation (Nygren et al., 2008). Cell fusion improved regeneration in rodent livers that received bone marrow transplants (Vassilopoulos et al., 2003; Wang et al., 2003). Myocardial infarction also triggers fusion events between cardiomyocytes and bone marrow transplanted cells (Oh et al., 2003; Figure 1). Similarly, human MSC may fuse to small airway epithelial cells upon insult *ex vivo* (Spees et al., 2003). However, to which extent mitochondrial transfer in these scenarios participates in the potential of stem cell fusion is unknown.

Cell fusion can modify the potential of the cells involved, having important implications in regeneration and cancer (Aguilar et al., 2013). A major concern is whether fusion is physiologically relevant, since most studies used pluripotent or multipotent stem cells as at least one of the fusion partners. It is difficult to evaluate its actual impact in tissues, since cytokinesis may occur after fusion events, leaving new synkaryons that could be hardly distinguished from the rest of the cells in the tissue. This is the underlying reason why *in vivo* reports of adult cell fusion are scarce, apart from those cells that form heterokaryons as part of their functional cycle, e.g., muscle, placenta or macrophages.

### Other cellular mechanisms

Mitochondrial extrusion is another possible mechanism for mitochondrial transfer. It allows the release of mitochondria or mitochondrial components from cells under specific conditions. During tumor necrosis factor α-induced cell death, cytoplasmic vacuoles engulf mitochondria, which subsequently fuse with the plasma membrane, releasing free mitochondria to the extracellular medium (Nakajima et al., 2008). Likewise, LPS stimulation promotes the fusion of autophagolysosomes with the plasma membrane and the release of mitochondrial components that activate the inflammatory response (Unuma et al., 2015). Neutrophils can extrude mitochondrial components, specifically oxidized mitochondrial nucleoids, which boost type I interferon secretion and can lead to the development of lupus (Caielli et al., 2016). Under high rates of reactive oxygen species (ROS) HeLa cells can extrude fragments of mitochondria (Lyamzaev et al., 2008). Basophils release mtDNA in ROS dependent manner to create extracellular DNA traps to fight bacteria (Morshed et al., 2014). Likewise, eosinophils rapidly release mtDNA in the extracellular space to bind and kill infectious bacteria (Yousefi et al., 2008). Mitochondrial extrusion not only occurs *in vitro*, but also *in vivo*. For example, platelets extrude functional mitochondria both encapsulated in microparticles and as free organelles. This way, they enhance inflammatory responses (Boudreau et al., 2014). In addition, hepatocytes of mice treated with anti-Fas antibody extruded mitochondria, which were detected in the perisinusoidal space and in serum (Nakajima et al., 2008).

## Future perspectives and therapeutic opportunities

Horizontal mitochondrial transfer among cells occurs *in vitro* and *in vivo*. However, whether mitochondria move between cells under physiological conditions, and the precise role of this transfer remain unknown. Stem cells are likely organelle donors; thus, adult stem cells, mesenchymal stromal cells, fibroblasts, and hematopoietic stem cells, emerge as potential mitochondrial donors. These cells may transfer mitochondria to surrounding cells in their niche, thus affecting cell differentiation, proliferation, tissue homeostasis, development and aging. During the course of an immune response, immune cells show high capacity to exchange surface receptors and intercellular material (Rechavi et al., 2009). These cells can also extrude mitochondrial components under specific conditions (Weinberg et al., 2015). Regarding the control of immune responses by those mechanisms, cognate immune cell-cell interactions such as immune synapses between lymphocytes and antigen-presenting cells, lymphocyte adhesion and migration across inflamed tissues or macrophage patrolling, may represent physiological scenarios in which mitochondrial intercellular exchange could control signaling, proliferation, bioenergetics or transcellular degradation of mitochondria. Professional phagocytic cells involved in inflammation and tissue regeneration may also acquire exogenous mitochondria (Phinney et al., 2015), as it has been observed in the optic nerve head (Davis et al., 2014). Notably, astrocytes transfer mitochondria to neurons supporting neuronal viability after ischaemic stroke (Hayakawa et al., 2016). Whether neurons and microglia crosstalk through intercellular mitochondrial transfer should be further addressed, in order to determine if defects on this type of communication are involved in pathogenesis and can represent a therapeutic target.

Unraveling the physiological significance of mitochondrial transfer requires the development of novel fluorescence and genetic mitochondrial tracking tools. Many of the studies referred here use fluorescent dyes to track mitochondrial transfer, but the possibility of the leakage of these dyes is a limitation for this methodology. Visualizing intercellular organelle transfer in real time in the intact living organism remains a major methodological challenge. The use of available cells and mice with fluorescent proteins confined to mitochondria will greatly accelerate the progress in this field (Abe et al., 2011; Pham et al., 2012). In addition, the use of mtDNA polymorphisms that allow the distinction of mtDNA from the donor and acceptor cells after mitochondria acquisition, the development of techniques to detect mitochondrial transfer (Caicedo et al., 2015) and low levels of cell heteroplasmy in single cells from tissues, will help to unequivocally ascertain whether mitochondrial transfer occurs in the different scenarios. Importantly, the possible exchange of healthy mitochondria to sustain cells with OXPHOS deficiencies would prevent the manifestation of some diseases associated with mitochondrial dysfunction or with the heteroplasmic inheritance of mutated mtDNA. Hence, additional study is required to evaluate the real impact of these mechanisms in mitochondrial diseases.

The number of diseases in which impaired mitochondrial function is thought to contribute to pathogenesis is currently increasing. While the main strategy employed in the treatment of mitochondrial dysfunction involves targeting mitochondrial genes encoded by the nuclear genome, our current capacity to supplement, repair, or replace mitochondrial DNA in somatic cells is very limited (Patananan et al., 2016).

Identification of the signaling pathways and the proteins controlling the transfer of mitochondria will improve its potential therapeutic application. Future therapeutic implications of this research need to consider strategies to pharmacologically enhance intercellular organelle transfer when desirable, or block its occurrence when it is deleterious, such as in the spread of infection or the maintenance of malignant cells. Given the considerable therapeutic potential of MSCs and mitochondrial transfer, future investigation of the mechanisms underlying the regulation of oxidative metabolism and dynamics of mitochondria in MSCs may ultimately facilitate the development of effective stem cell therapies for treatment of mitochondrial diseases. Elucidation of the mechanisms of mitochondrial transfer will help us create a potential cell therapy-based mitochondrial restoration or mitochondrial gene therapy for human diseases caused by mitochondrial dysfunction.

Another major concern pertains the required transfer dose. It might not be necessary to fully replace the mitochondria repertoire in the recipient cell to restore mitochondrial dysfunction. It is likely that the transfer of a low number of copies of a functional mtDNA could restore the normal copy number and therefore the mitochondrial function. Hence, tumors formed from cells with low copy number of mtDNA are able to recover them after injection into immunoincompetent mice (Dickinson et al., 2013). This would open a new field in which mitochondrial transfer therapy would be an option to treat injured tissues, taking into account that using healthy mitochondria from the same patient could lead to a better performance of the tissues because of the matching between mitochondrial and nuclear DNA (Latorre-Pellicer et al., 2016).

## Author contributions

DT, FB, and FS: Elaboration and writing of the manuscript and making Figures. FS and FB contributed equally to this work.

### Conflict of interest statement

The authors declare that the research was conducted in the absence of any commercial or financial relationships that could be construed as a potential conflict of interest. The reviewer RG and handling Editor declared their shared affiliation, and the handling Editor states that the process nevertheless met the standards of a fair and objective review.
